# Nonalcoholic Steatohepatitis (NASH) and Atherosclerosis: Explaining Their Pathophysiology, Association and the Role of Incretin-Based Drugs

**DOI:** 10.3390/antiox11061060

**Published:** 2022-05-27

**Authors:** Eleftheria Galatou, Elena Mourelatou, Sophia Hatziantoniou, Ioannis S. Vizirianakis

**Affiliations:** 1 Department of Life & Health Sciences, School of Sciences and Engineering, University of Nicosia, 2417 Nicosia, Cyprus; ivizir@pharm.auth.gr; 2 Laboratory of Pharmaceutical Technology, Department of Pharmacy, School of Health Sciences, University of Patras, 26504 Patras, Greece; sohatzi@upatras.gr; 3 Laboratory of Pharmacology, School of Pharmacy, Aristotle University of Thessaloniki, 54124 Thessaloniki, Greece

**Keywords:** nonalcoholic steatohepatitis (NASH), nonalcoholic fatty liver disease (NAFLD), atherosclerosis, inflammation, oxidative stress, GLP-1 RAs, DPP4-i, incretin-based drugs, cardiovascular outcome trial (CVOT), lipotoxicity

## Abstract

Nonalcoholic steatohepatitis (NASH) is the most severe manifestation of nonalcoholic fatty liver disease (NAFLD), a common complication of type 2 diabetes, and may lead to cirrhosis and hepatocellular carcinoma. Oxidative stress and liver cell damage are the major triggers of the severe hepatic inflammation that characterizes NASH, which is highly correlated with atherosclerosis and coronary artery disease. Regarding drug therapy, research on the role of GLP-1 analogues and DPP4 inhibitors, novel classes of antidiabetic drugs, is growing. In this review, we outline the association between NASH and atherosclerosis, the underlying molecular mechanisms, and the effects of incretin-based drugs, especially GLP-1 RAs, for the therapeutic management of these conditions.

## 1. Introduction

Nonalcoholic fatty liver disease (NAFLD), the most common chronic liver disease, encompasses a wide spectrum of pathologies ranging from simple steatosis (hepatic lipid accumulation) to nonalcoholic steatohepatitis (NASH), which can progress to severe liver damage, cirrhosis, and end-stage liver diseases, including hepatocellular carcinoma [1]. Robust evidence from epidemiological and familial studies demonstrates the elevated heritability of liver steatosis and NAFLD pathogenesis and progression to NASH. Concepts in the literature have proposed a multi-hit model to describe the pathophysiology of NASH, demonstrating that NASH results from a culmination of various factors in parallel, including altered lipid metabolism, oxidative stress, endoplasmic reticulum (ER) stress, altered production of adipokines and cytokines, mitochondrial dysfunction, lipotoxicity, gut-derived endotoxin, and genetic predisposition [2,3,4].

The prevalence of NAFLD in developed countries is estimated to be between 20% and 30%, while the prevalence of NASH is about 3–5% [5]. However, in obese patients and patients with type 2 diabetes the prevalence of NAFLD rises up to 90% and 75%, respectively [6]. Although people with high BMIs are at greater risk of fatty liver complications, individuals with normal BMIs are also diagnosed with NAFLD and are categorized as lean NAFLD. Liver diseases could be prevented and treated, reducing the ratio of premature morbidity and mortality, if measures for prevention and early detection are properly implemented, according to the European Association for the Study of the Liver (EASL)–Lancet Liver Commission [7].

NAFLD/NASH is manifested as hepatic metabolic syndrome (MetS), a highly atherogenic condition, even at a very early age, as a consequence of metabolic imbalance but also can act as a trigger for further metabolic abnormalities and is strongly associated with obesity, type 2 diabetes, and insulin resistance, leading to a high risk of cardiovascular diseases (CVDs) [8].

Currently, there are no drug therapies approved by the Food and Drug Association (FDA) for the treatment of NAFLD [9]. Treatment options are mainly centered around lifestyle changes (e.g., weight loss, reduced caloric intake, and exercise) and, in some cases, the administration of Vitamin E or pioglitazone [10]. However, various drugs are being clinically evaluated for their effectiveness in NAFLD/NASH, especially antidiabetic drugs since type 2 diabetes mellitus (T2DM) has a close association with NAFLD/NASH development and progression. Amongst these drugs, incretin-based drugs, especially glucagon-like peptide-1 receptor agonists (GLP-1 RAs), have been found to exert a beneficial role, with multiple mechanisms being involved since GLP-1 receptors can be located in various body tissues [11].

CVDs represent the main cause of mortality in patients with NAFLD (40–45% of total deaths), followed by extrahepatic malignancies and liver-related complications. Moreover, patients with NASH were reported to have a much higher incidence of coronary artery disease-related mortality, thus indicating that NASH contributes actively to the pathogenesis of atherosclerosis [12]. Cardiovascular outcome trials (CVOTs) that have been conducted in GLP-1 RAs, as part of their safety assessment, have demonstrated that several of the drugs belonging to this group can exhibit beneficial effects in CVDs by preventing the occurrence of major atherogenic CV events [13].

In this review, we summarize the current knowledge on the pathophysiology of both NAFLD/NASH and atherosclerosis, focusing on the mechanisms linking NASH and atherosclerosis development and progression. Based on these similarities, we validate the hypothesis that NASH and atherosclerosis share key pathophysiological characteristics, with a common etiology being inflammation and oxidative stress involving activated macrophages. Moreover, the potential of incretin-based drugs, especially GLP-1 RAs, in treating NASH and atherosclerosis is discussed. For this purpose, the clinical data demonstrating the effects of these drugs in both NASH and atherosclerotic cardiovascular events as well as data suggesting the potential mechanisms exerted by these drugs, which are related to the management of both NASH and atherosclerosis, are provided.

## 2. Diagnosis of NAFLD/NASH

NAFLD comprises a wide spectrum of liver diseases ranging from simple fat accumulation to more advanced stages, including NASH, cirrhosis, and cancer. The diagnosis of NAFLD requires the identification of hepatic steatosis (liver fat > 5%) in the absence of other causes of liver fat accumulation (e.g., alcohol consumption or coexisting causes of chronic liver diseases) [9,14]. NAFLD is often suspected in clinical practice when an individual presents features of MetS, such us abdominal obesity, hypertension, increased levels of triglycerides, low levels of HDL, and increased levels of fasting blood glucose. In the same context, individuals with persistently abnormal liver enzyme levels should be screened for NAFLD/NASH [9]. Several studies have shown that mild-to-moderate elevations in serum liver enzymes or increased liver volume [15] are associated with a higher risk of all-cause mortality [16,17,18]. There are many available imaging techniques, including ultrasound, image-guided biopsy, computed tomography (CT), and magnetic resonance imaging (MRI). Multiparametric MRI combines two or more quantitative techniques, such as T1, T2, and the proton density fat fraction (PDFF), to assess hepatic inflammation and fibrosis with a high level of accuracy. MRI-PDFF was more accurate at detecting changes in liver fat than liver biopsy and has been validated in multiple studies [19,20,21]. Ultrasound is widely accepted as a first-line diagnostic tool since it is a non-invasive, low cost, and radiation-free technique with a satisfactory sensitivity for moderate and severe steatosis identification [22]. To date, the gold standard to diagnose patients with NASH is still considered to be liver biopsy, demonstrating the typical fibrosis pattern, which cannot be seen via imaging methods. Currently, although there is no readily available, reliable, and non-invasive method to identify the progression of steatosis to NASH and fibrosis, altered levels of lipoprotein (a) (Lp(a)) [23] and several biomarkers of inflammation (such as ferritin and high-sensitivity C reactive protein (CRP)) and apoptosis (cytokeratine 18 (CK-18)) [24] have been associated with the diagnosis of NASH in NAFLD patients. Moreover, the NAFLD Activity Score (NAS) is used in clinical trials for evaluating the changes in histological features caused by therapeutic interventions. This system was developed and validated by the Pathology Committee of the NASH Clinical Research Network (NASH CRN) and is based on the semi-quantitative evaluation of histological features, specifically steatosis (0–3), lobular inflammation (0–2), and hepatocellular ballooning (0–2). NAS is calculated by adding these values, with a sum of ≥5 indicating NASH and scores < 3 considered as “not NASH” [25]. However, the threshold value of 5 in NAS is not always in accordance with a NASH diagnosis that is based on the analysis of a liver biopsy for the existence of certain lesions with specific patterns [26].

## 3. Genetic Factors Involved in NAFLD/NASH

Multiple genome-wide association studies (GWAS) and large candidate gene studies have addressed the contribution of genetic factors in NAFLD and the progression to NASH and fibrosis [27,28]. The dominant genetic modifiers of NAFLD susceptibility and progression are the variants in patatin-like phospholipase domain-containing 3 (PNPLA3), transmembrane 6 superfamily member 2 (TM6SF2), membrane-bound O-acyltransferase domain-containing 7 (MBOAT7), glucokinase regulator (GCKR), and hydroxysteroid 17-beta dehydrogenase 13 (17β-HSD13) genes. The PNPLA3 polymorphism rs738409 C>G encoding for the I148M protein is the most robust and well-replicated genetic variant associated with NAFLD [27], alcoholic fatty liver disease (AFLD), NASH [29], and severe alcohol-related steatohepatitis (ASH) [28] and is related to TG accumulation, lipid droplet remodeling, and lipotoxicity [30]. The genetic variant rs58542926 C>T in the TM6SF2 gene is associated with higher circulating levels of ALT, hepatic TG content, and NAFLD progression [31]. Moreover, the rs641738 C>T variant in the MBOAT7 gene as well as the loss-of-function rs1260326 C>T variant in the GCKR gene are related to inflammation, increased susceptibility to NASH, fibrosis, and hepatocellular carcinoma (HCC) [30]. Variants of 17β-HSD13 are associated with increased steatosis but decreased inflammation and lower ALT levels in NAFLD [32]. Recently, a GWAS identified a new signal on chromosome 15 (rs11858624) in the Pygopus family PHD finger 1 (PYGO1) gene, a novel steatosis modifier that contributes to the Wnt signaling pathway, suggesting that Wnt signaling pathways may be relevant in NAFLD/NASH pathogenesis [33].

An altered miRNA profile [34] as well as epigenetic changes in key regulators of mitochondrial biogenesis, fatty acid oxidation, oxidative stress, inflammatory, and fibrotic pathways have been described in patients with NAFLD and NASH [30].

However, advanced liver fibrosis in NASH is often accompanied by a reduction in hepatic fat to the point of complete fat loss (burnt-out NASH). This paradox can be partially explained by the identification of somatic mutations in genes involved in the regulation of lipid metabolism (forkhead transcription factor O1, FOXO1; cell-death-inducing DFFA-like effector b, CIDEB; and glycerol-3-phosphate acyltransferase, GPAM) in patients with advanced NAFLD [35].

Finally, besides the genetic factors, sex differences play a key role in the development of NAFLD/NASH. In general adult populations, the overall NAFLD/NASH prevalence is higher in men than in women and becomes similar after the age of 50–60 years [36]. After menopause, NAFLD occurs at a higher rate in women, suggesting the protective role of estrogen. Another study demonstrated that premenopausal women with NAFLD had more severe lobular inflammation and hepatocyte ballooning than men or postmenopausal women, suggesting that female hormones modulate hepatic injury and inflammation [37].

## 4. Pathophysiological Mechanisms of NAFLD and NASH

NAFLD is a metabolic disorder, and its pathogenesis is a multifactorial process that involves a complex interaction between metabolic, clinical, environmental, and genetic factors [38,39].

### 4.1. Nutrition and Gut Microbiota

Components of nutrition and caloric intake play a key role in NAFLD development and progression. A nutritional high-fat and hypercaloric pattern rich in saturated fat and omega-6 (n-6) polyunsaturated fatty acids (PUFAs), carbohydrates, and low amounts of omega-3 (n-3) PUFAs and fibers have all been associated with NAFLD [40]. Data from preclinical and clinical studies demonstrate that fructose intake in hypercaloric diets is associated with an increased intrahepatic content of triglycerides, de novo lipogenesis, hepatic steatosis, obesity, and insulin resistance [41,42]. Glucose intake exerts similar detrimental effects on liver health by increasing hepatic lipid accumulation in healthy men [43]. This energy imbalance leads to higher post-prandial blood glucose levels and, thus, a higher insulin secretion rate. Insulin further stimulates de novo lipogenesis, leading to hepatic inflammation and subsequent NASH development. It has been demonstrated that a high-fat diet induces hepatic tumor necrosis factor-a (TNF-a) and interleukin (IL)-6 expression, whereas their inhibition prevents hepatic steatosis and NAFLD progression.

The body of evidence supporting an association between gut microbiota and NAFLD pathogenesis and progression is increasing. Basic players for complex dietary carbohydrate degradation are gut bacteria, leading to the production of short-chain fatty acid (SCFA) metabolites, including acetate, propionate, and butyrate. Although these metabolites improve glucose and lipid metabolism and maintain intestinal homeostasis, the increased production may contribute to obesity and liver steatosis, enhancing nutrient absorption [44]. Moreover, bile acids, which are synthesized by cholesterol within the liver, metabolized in the small intestine, and reabsorbed back to the liver through the portal vein, are significant regulators of lipid and carbohydrate metabolism and, subsequently, energy homeostasis. In the liver, they act as signaling molecules through their binding and activation of several nuclear hormone receptors, including farnesoid X receptors (FXRs) and G-protein-coupled receptor 5 (TGR5) [45]. Many studies have assessed the interplay between bile acids and microbiota, demonstrating that bile acids regulate and control the microbiome, while gut bacteria contribute to several biotransformations of bile acids and affect their composition, modulating hepatic steatosis [46,47,48,49].

It is well-documented that patients with biopsy-proven NAFLD/NASH exhibit a different microbiota signature and increased gut permeability due to a disruption of gut epithelial tight junctions. The leaky intestine leads to a translocation of bacteria and bacteria-produced endotoxins and alcohol through the portal circulation to the liver, contributing to ROS generation, hepatic inflammation through the toll-like receptor (TLR)-4-mediated pathway, and possibly fibrogenesis [49,50,51].

### 4.2. Adipose Tissue Dysfunction—The Role of Adipokines

Adipose tissue is an essential and highly active metabolic and endocrine organ that stores triacylglycerol as an energy source and releases adipokines and cytokines that are suspected to play a key role in NAFLD development and the progression to NASH. Visceral adiposity leads to excessive lipid accumulation and is highly correlated with insulin resistance due to an imbalance in pro-inflammatory and anti-inflammatory cytokine release. Leptin, an adipokine that plays a crucial role in the regulation of body weight and fat content, primarily acts centrally to reduce food intake, increase energy expenditure, and prevent lipid accumulation in organs other than the adipose tissue. However, excessive levels of leptin may result in hepatic inflammation and fibrosis [52]. Adiponectin, an abundant adipokine with anti-inflammatory and antifibrotic properties that acts on Kupffer cells and hepatic stellate cells (HSCs), increases hepatic insulin sensitivity by suppressing gluconeogenesis and lipogenesis and reduces body fat [50]. The anti-inflammatory effects of adiponectin are achieved by: (1) the suppression of transcription factor NF-κB, (2) the inhibition of pro-inflammatory cytokine release, and (3) the stimulation of anti-inflammatory cytokine secretion [51]. Patients with NAFLD and NASH exhibit elevated leptin levels and decreased adiponectin levels, which are associated with the severity of NAFLD patients, probably reflecting the increasing insulin resistance [8,52]. However, in later stages of NASH progression to cirrhosis, adiponectin levels are increased, possibly due to an impaired clearance of adiponectin and an excessive release of pro-inflammatory cytokines.

### 4.3. Insulin Resistance and Hepatic Fat Accumulation

Two pivotal characteristics of NAFLD pathophysiology are insulin resistance and hepatic steatosis. Insulin plays a crucial role in the regulation of glucose and lipid metabolism in several metabolic tissues, including adipose tissue and the liver. In hepatocytes, insulin regulates glucose uptake, promotes glycogenesis, and activates key regulators of de novo lipogenesis (DNL) while it simultaneously decreases gluconeogenesis, promoting glycogen storage [53]. On the other hand, in adipocytes, insulin has three main actions: (1) to promote the esterification of fatty acids, (2) to promote the storage of esterified fatty acids, including triglycerides (TGs), in lipid droplets, and (3) to inhibit lipolysis via hormone-sensitive lipase inactivation [54].

In an obese state and in NAFLD patients, systemic insulin resistance results in increased lipolysis and, thus, excess free fatty acids (FFAs), and inflammatory cytokines from peripheral adipose tissue can enter the liver through the portal circulation. The accumulation of FFAs and lipid metabolites in hepatocytes may induce a disruption of the insulin-signaling pathway and, subsequently, hepatic insulin resistance. Moreover, hepatic insulin resistance contributes to hyperglycemia, hyperinsulinemia, and increased lipid accumulation through DNL stimulation and mitochondrial fatty acid β-oxidation (FAO) inhibition, thus aggravating hepatic steatosis [55,56].

### 4.4. Progression of NAFLD to NASH

#### 4.4.1. Lipotoxicity and Oxidative Stress

Several studies have highlighted that lipotoxicity leads to hepatocyte injury and the progression of NASH. The imbalance between lipid acquisition (increased uptake of circulating FFAs and DNL) and lipid exportation (downregulated FAO and export of lipids in very low density lipoproteins (VLDL)) further promotes lipid accumulation in the liver and the progression of hepatic steatosis [56,57,58]. In addition to FFAs, other types of lipids and their derivatives, including free cholesterol and ceramides, are involved in the development of liver lipotoxicity in NAFLD and NASH patients [59,60]. Lipotoxicity-induced hepatic injury leads to hepatocyte ballooning degeneration (liver cell swelling), fibrosis, and glomerular inflammation, which are considered to be the key histological features for NASH diagnosis [61,62].

In NAFLD patients, mitochondrial dysfunction plays a pivotal role during the transition from simple steatosis to NASH [63]. The energy homeostasis in hepatic cells is regulated by mitochondrial FAO, electron transfer, the production of ATP, and reactive oxygen species (ROS) [64]. Mitochondrial dysfunction contributes to an imbalance between prooxidant and antioxidant mechanisms, thus leading to lipid accumulation and excess ROS generation. The latter leads to the activation of inflammatory mediators and signaling pathways exacerbating inflammation, ROS generation, and oxidative DNA damage in NASH patients [65,66]. The overload of FFAs leads to an increase in the permeability of the inner mitochondrial membrane, 31–40% lower maximal respiration associated with mitochondrial uncoupling, electron leakage, augmented hepatic oxidative stress, and oxidative DNA damage [4,65].

#### 4.4.2. Hepatic Inflammation and Fibrosis

The mechanistic concepts of inflammation in NAFLD/NASH have recently been reviewed and are associated with the exacerbated production of inflammatory factors from extrahepatic tissues (adipose tissue and gut) and in the liver by injured hepatocytes and the activation of resident hepatic macrophages, Kupffer cells [67]. Excess levels of circulating and hepatic FFA accumulation, altered gut microbiome, gut permeability alterations, the release of endotoxin, and adipose tissue dysfunction lead to hepatocyte injury and apoptosis, ROS generation, and inflammatory response, representing the initial steps of progression to NASH. Immunogenic stimuli, including damage-associated molecular patterns (DAMPs) released by injured hepatocytes and pathogen-associated molecular patterns (PAMPs), are recognized by the innate immune system through pattern recognition receptors (PRRs), such as toll-like receptors (TLRs) and NOD-like receptors (NLRs), and play a key role in NASH [68]. Being widely expressed in hepatic cells, activated TLRs, particularly TLR-4, recruit Kupffer cells, which release pro-inflammatory cytokines, including TNF-a, IL-1, IL-6, fibrogenic factors such as TGF-β, and the further activation of pro-inflammatory transcription factors (NF-κB) [69]. Cytokine signaling promotes the recruitment of immune effector cells, including neutrophils, dendritic cells, natural killer (NK) cells, and cytotoxic T cells, with subsequent hepatocyte injury via oxidative-stress-mediated mechanisms, indicating that adaptive immunity plays an important role in the progression of this disease [70]. In NASH patients, NOD-like receptor protein 3 (NLRP3) inflammasome is upregulated in injured hepatocytes, Kupffer cells, and liver sinusoidal endothelial cells and can be activated by DAMPs and PAMPs [71,72].

Chronic inflammation is associated with fibrosis, which can further progress to bridging fibrosis and cirrhosis. In response to liver injury, activated Kupffer cells, infiltrating monocytes, activated and aggregated platelets, and damaged hepatocytes release platelet-derived growth factor (PDGF) and TGF-β1, leading to HSC activation. Upon their activation, HSCs express NLRP3 inflammasome and transdifferentiate into fibroblasts or myofibroblast-like cells with proliferative, inflammatory, and migratory properties [73]. Upon HSC proliferation, components of the extracellular matrix are produced, including collagen type I and type III as well as tissue inhibitor of metalloproteinases 1 (TIMP-1), all contributing to fibrogenesis [74,75].

## 5. Association between NASH and Atherosclerosis: Inflammation and Oxidative Stress as Key Players

A large body of epidemiological and clinical evidence demonstrates that NAFLD is not only associated with liver morbidity but also with CVD development, arrhythmias, left ventricular dysfunction, and heart failure [76,77,78]. NAFLD and CVD are both manifestations of end-organ damage of MetS and share several common environmental and genetic factors [79,80,81] It has been demonstrated that NAFLD progression to NASH is associated with more severe atherosclerosis and is even considered an independent risk factor for coronary artery disease (CAD) [8,82]. Likewise, atherosclerosis accompanied by NAFLD/NASH has more adverse metabolic burdens than atherosclerosis alone. In this aspect, several studies provide evidence of a strong association between NASH and: (1) carotid atherosclerosis [83,84,85,86] and (2) subclinical manifestations of atherosclerosis in patients with or without T2DM, including increased intima–media thickness, endothelial dysfunction, arterial stiffness, impaired left ventricular function, reduced flow-mediated vasodilation, and coronary calcification [86,87,88]. These associations are independent of traditional cardiovascular risk factors and MetS characteristics across a wide range of patient populations [89].

Atherosclerosis is a progressive multifactorial disease characterized by the thickening of the arteries and endothelial dysfunction and is a main cause of myocardial infarction and stroke [90,91] Proinflammatory activation of endothelial cells (ECs) initiate the penetration of monocytes into the intima media, where they predominantly mature to pro-inflammatory macrophages (the M1 phenotype) that actively take up modified low-density lipoproteins (oxLDL) via scavenger receptors (e.g., scavenger receptor A1 (SR-A1), lectin-like oxLDL receptor-1 (LOX-1), and CD36) and release a variety of inflammatory cytokines and chemokines that are essential for the propagation of inflammation [92]. The excessive influx of modified LDLs and the accumulation of cholesterol esters in intimal macrophages due to the increased vascular permeability of the endothelial barrier lead to the generation of foam cells, which play a key role at all stages of atherosclerotic lesion development, from initial vascular lesions to advanced plaques [91]. Another main imbalance observed in atherosclerosis is the upregulation of acetyl-CoA acetyltransferase (ACAT1), the enzyme responsible for cholesterol esterification, and the downregulation of neutral cholesterol ester hydrolase (NCEH), the enzyme responsible for the hydrolysis of cholesterol esters to free cholesterol, resulting in the accumulation of cholesterol esters and the further transformation of macrophages to foam cells. Yellow foam cells aggregate on the arterial walls and cause the development of fatty streaks, which form a fibrous atherosclerotic plaque cap [93,94].

At advanced stages of the disease, growth factor released by macrophages in the plaque causes the proliferation of smooth muscle cells and the plaque becomes fibrotic [95]. Activated macrophages and T lymphocytes of the fibrous atherosclerotic plaque cap stimulate the production of proteolytic metalloproteases, leading to the degradation of the extracellular matrix by phagocytosis and to a decrease in the stability of the fibrous cap. Plaque rupture leads to a coagulation process, blood clot formation, thrombus formation, and a blockade of the arteries [96]. These atherogenic processes are triggered by well-identified risk factors, such as hypertension, hyperlipidemia, and diabetes mellitus.

**Figure 1:**
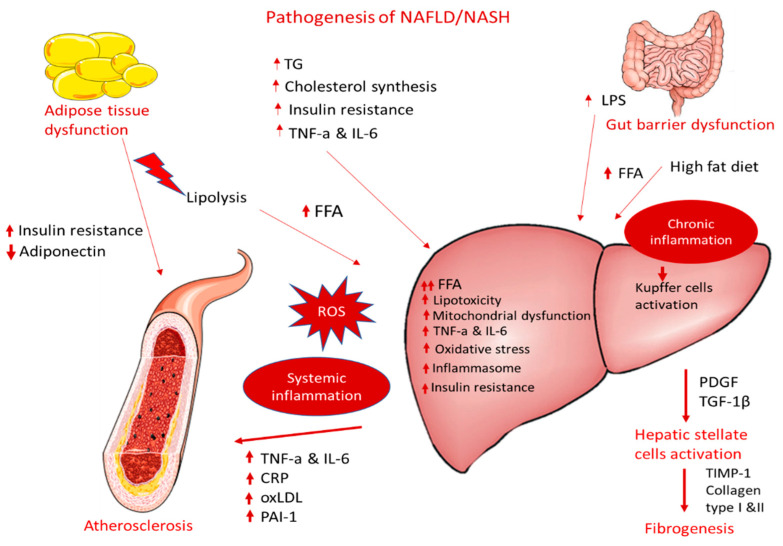
Pathogenesis of NAFLD/NASH and association with atherosclerosis.

NAFLD can contribute to and aggravate atherosclerosis development, but the precise mechanisms remain unclear. The supposed mechanisms for accelerating atherosclerotic disease in patients with NASH are very complex and include, among others, chronic inflammation, lipid accumulation, and oxidative stress (Figure 1). The following sections display possible linkages between these conditions at the molecular level.

### 5.1. Dyslipidemia and Lipotoxicity

As mentioned before, NAFLD/NASH is characterized by hepatic fat accumulation, which results from an imbalance between lipid acquisition and lipid disposal that is mediated by increased hepatic DNL and the uptake of circulating FFAs, a downregulation of compensatory FAO, and an altered export of lipids in VLDL. This imbalance is considered to be the initiating mechanism of atherosclerosis as well [97]. Moreover, patients with NAFLD exert typical atherogenic dyslipidemia features, including higher serum TG and oxLDL levels and lower serum HDL. Moreover, high serum oxLDL levels are more dominant in patients with atherosclerosis due to their localization in macrophage-derived foam cells. It has been reported that TNF-a is implicated in the decrease in HDL levels, indicating a link between inflammation and the development and progression of insulin-resistant conditions, including NASH and atherosclerosis [98,99].

### 5.2. Adipose Tissue Dysfunction

Adipose tissue increases CVD risk by inducing many obesity-associated complications, such as dyslipidemia, high blood pressure, insulin resistance, and T2DM [100]. Visceral adiposity causes an increase in the hepatic accumulation of FFAs, accompanied by decreased FFA oxidation and altered glucose metabolism, contributing to hepatic insulin resistance. Visceral obesity is associated with proinflammatory cytokine production, i.e., TNF-α, IL-6, monocyte chemoattractant protein-1 (MCP-1), CRP, adipokines, and macrophage infiltration, resulting in local and systemic inflammation. The latter is associated with the consequent hepatic production of pro-atherogenic molecules, such as plasminogen activator inhibitor-1 (PAI-1) and fibrinogen, thereby causing endothelial dysfunction and increasing the risk of atherothrombosis.

A consistent and ever-growing line of research has revealed a link between intestinal dysbiosis, inflamed adipose tissue, and NAFLD with atherosclerosis and other cardiac complications. The progression of NAFLD to NASH leads to the production of proinflammatory cytokines, atherogenic lipoproteins, and vasoactive and thrombogenic factors and enhanced oxidative stress, resulting in an increased risk of atherosclerosis and myocardial infarction.

### 5.3. Endothelial Dysfunction and Inflammation

The endothelial dysfunction and abnormal vasoreactivity observed in NASH patients lead to chronic inflammation, increased vasoconstriction, and increased prothrombotic factor production, thus elevating the risk of atherosclerosis and several other cardiovascular implications [101].

Inflammation plays a central role in both NASH and atherosclerosis, involving the local presence or resident macrophages. Macrophages accumulate oxidized lipoproteins through the SRs and lead to the generation of foam cells and the release of cytokines during atherogenesis. Similarly, in NASH the resident hepatic macrophages, Kupffer cells, take up modified lipoproteins expressing SR CD36 and, thus, further contribute to atherosclerotic lesions [93,99].

Specifically, immunogenic stimuli, including the accumulation of modified triglyceride-rich lipoproteins, serve as DAMPs and activate TLRs recruiting macrophages and resident Kupffer cells. The activated NLRP3 inflammasome leads to the release of pro-inflammatory cytokines, including TNF-a, IL-1, and IL-6; fibrogenic factors, such as TGF-β; and the further activation of pro-inflammatory transcription factors (NF-κB) [69], providing an important link between NASH, liver fibrosis, and the development of vascular damage and atherosclerosis [102].

## 6. Treatment Options for NASH

Based on the current guidelines available from the American Association for the Study of Liver Diseases (AASLD) (2018), the EASL (2016), and The Asia-Pacific Working Party on NAFLD (2017), the management of NAFLD is mainly focused on lifestyle interventions, primarily involving loss of weight (7–10%) achieved through controlled caloric intake and moderate-intensity exercise. In terms of specific diet recommendations, only EASL recommends following a Mediterranean diet and avoiding processed foods and added fructose that can promote NAFLD. In terms of pharmacological interventions, many drugs are being clinically evaluated for the treatment of NAFLD or NASH, but so far, no drug has received approval. The administration of vitamin E is recommended by AASLD for patients diagnosed with NASH based on biopsy results as long as they are not suffering from diabetes or cirrhosis. The administration of pioglitazone is recommended by AASLD and the Asia-Pacific Working Party on NAFLD in patients with NASH with or without diabetes, with the recommendation of only short-term usage of the drug. On the other hand, EASL recommends drug usage only in NASH patients with fibrosis or an with increased risk for progression of the disease (T2DM, MetS, or increased levels of alanine aminotransferase (ALT)) without specific guidelines on the usage of Vitamin E or pioglitazone [10]. Since only a small percentage of patients (10–20%) can successfully implement the proposed lifestyle changes in the long run, the necessity of effective medications for the management of NASH is evident [11]. Moreover, since most patients with NAFLD have a high risk to develop atherosclerotic cardiovascular diseases, which in turn are the most common cause of death for these patients, treating atherosclerosis along with NAFLD/NASH is crucial for an improved prognosis.

Amongst the pharmacotherapies that are currently clinically evaluated for the treatment of NASH are incretin-based drugs, such as glucagon-like peptide-1 receptor agonists (GLP-1 RAs) and dipeptidyl dipeptidase-4 inhibitors (DPP-4i), drugs that are mainly used as antidiabetics. T2DM has a close association with NASH, especially in terms of insulin resistance. Hence, these antidiabetics have been tested for their effectiveness in NAFLD and NASH as well. An analysis of observational data in T2DM patients demonstrated that a high percentage of them (approximately 70%) exhibited liver steatosis, and the administration of GLP-1 RAs led to steatosis reduction over the course of 24 months [103]. There are two groups of GLP-1 RAs, depending on their duration of action, the short-acting (lixisenatide once daily and exenatide twice daily) and the long-acting (semaglutide, liraglutide, dulaglutide, albiglutide, and exenatide once weekly) GLP-1 RAs, which differ in pharmacodynamics and pharmacokinetics. In terms of pharmacodynamics, short-acting GLP-1 RAs’ main action is slowing down gastric emptying, a process that contributes to decreasing postprandial glucose, whereas long-acting GLP-1 RAs enhance the secretion of insulin and inhibit the secretion of glucagon (both postprandial and in the fasting state), thus reducing the levels of glucose. Moreover, long-acting GLP-1 RAs are better tolerated in the gastrointestinal system, have reduced administration frequencies, which is more convenient for patients and improves medication adherence, and provide more stable plasma concentrations [11].

## 7. Effectiveness of Incretin-Based Drugs on NASH and Atherosclerosis

### 7.1. GLP-1 RAs and DPP-4i Effectiveness on NASH

Several clinical trials have been performed to study the effect of GLP-1 RAs on NAFDL and NASH since 2007, most of which concerned the determination of the role of these drugs in NAFLD and recruited individuals with pre-existing T2DM. Fewer studies concerned the resolution of NASH. This is attributed to the more invasive methods required for NASH diagnosis as well as reliable evaluation of NASH progression, with liver biopsy being the ‘golden standard’, which increases the difficulty in recruiting individuals [104]. The performed studies varied in duration as well as in the selected endpoints. In most of the cases, biochemical markers (e.g., ALT) and fibrotic markers (e.g., ferritin) as well as imaging techniques for the evaluation of hepatic fat content (MRI, CT, transient elastography (FibroScan), and ultrasonography) and the NAS were used in order to assess the effect of GLP-1 RAs in hepatic steatosis or fibrosis [105]. Liver biopsy was used less frequently.

Based on results obtained from clinical trials conducted on the effect of incretin-based drugs on NASH resolution, only liraglutide and semaglutide (two long-acting GLP-1 RAs) were found to be beneficial [106]. The effect of liraglutide in NASH resolution was studied in the phase 2 LEAN clinical trial (liraglutide efficacy and action in non-alcoholic steatohepatitis), where overweight patients with clinical evidence of NASH were treated for 48 weeks with 1.8 mg/day of liraglutide administered subcutaneously. The results obtained after liver biopsy demonstrated that NASH was resolved at a 39% rate compared to 9% in the placebo group (relative risk 4.3; 95% CI, 1.0–17.7; *p* = 0,019). Moreover, a very low percentage of patients showed progression in fibrosis (9% of patients under treatment with liraglutide compared to 36% of patients receiving placebo) (relative risk 0.2; 95% CI, 0.1–1.0; *p* = 0.04). Specifically, steatosis and hepatocyte ballooning were improved, indicating less histological damage, although lobular inflammation and the overall NAFLD score remained the same. The latter was not significant in the prediction of morbidity or mortality related to liver function, which is greatly associated with NASH and fibrosis. Moreover, the trial indicated that liraglutide had a significant effect on the levels of serum γ-glutamyl transferase and glycated hemoglobin (HbA_1(c)_) as well as causing a significant reduction in body weight. No significant differences could be observed in the serum aminotransferase levels, HDL concentration, or systolic blood pressure. The overall results were attributed to the drug’s ability to control glucose levels as well as body weight, in combination with more direct actions on hepatocytes. These actions were indicated through in vitro experiments and are related to changes in the oxidation of fatty acids, de novo lipogenesis, and the transportation of lipids, which are all elements of NASH pathogenesis [107].

Semaglutide’s effect on NASH was investigated in a phase 2 clinical trial where three different daily doses administrated subcutaneously (0.1 mg, 0.2 mg, or 0.4 mg) were compared to placebo in terms of NASH resolution (primary endpoint) without a worsening of fibrosis (secondary endpoint). The study, which had a total duration of 72 weeks, demonstrated that the best results were obtained when the higher dose (0.4 mg/day) was administered (59% of patients with NASH resolution without a worsening of fibrosis compared to 17% in the placebo group; odds ratio 6.87; 95% CI, 2.60–17.63; *p* < 0.001). No significant improvement in fibrosis was observed (43% of patients with improved fibrosis in the group of patients receiving 0.4 mg/day compared to 33% in the placebo group; odds ratio, 1.42; 95% CI, 0.62–3.28; *p* = 0.48). Significant weight loss was observed, especially in the group receiving 0.4 mg/day (−13%), as well as reductions in HbA_1(c)_, alanine aminotransferase levels, and TGs [108].

The fact that both liraglutide and semaglutide showed no significant improvement in fibrosis in all the clinical trials conducted so far could be attributed to the duration of the studies, with longer periods being required to improve liver fibrosis, especially for patients that were originally in more advanced fibrosis states [106,109]. Currently, a phase 3 clinical trial on semaglutide (2.4 mg administered subcutaneously once weekly) with a longer duration (240 weeks) has started to evaluate the resolution of NASH with no worsening of liver fibrosis, the improvement in liver fibrosis with no worsening of steatohepatitis, and the time to the first liver-related clinical event (NCT04822181) (Table 1).

DPP-4 inhibitors (i.e., alogliptin, linagliptin, saxagliptin, sitagliptin, and vildagliptin), that are able to inhibit the enzyme dipeptidyl peptidase (DPP) IV and lead to increased levels of GLP-1 are drugs that are approved to treat T2DM. The available clinical data regarding the efficiency of DDP-4 inhibitors in NAFLD/NASH are limited and, in some cases, contradictory. The DPP-4 inhibitor that has attracted most of the research attention, with several clinical trials having been conducted, is sitagliptin. Most of the clinical trials with sitagliptin (50–100 mg/day) were performed in a small number of patients with T2DM (7–72) with durations ranging from 12 to 60 weeks. Out of the conducted clinical trials, only two open-label trials indicated a beneficial effect on biopsy-proven NASH with improvements in hepatocellular ballooning and NAS. Sitagliptin was administered at 100 mg/day for 1 year, but the number of patients that participated in these clinal trials was low (15–40 patients) [110,111]. These results could be attributed to suppressing oxidative and inflammatory processes in the liver exerted due to an increase in GLP-1 levels, as indicated in mouse models [112,113]. However, in another open-label clinical trial on biopsy-proven NASH patients (100 mg/day, 24 weeks, 12 patients) no reduction in hepatocellular ballooning, NAS score, liver fibrosis, lobular inflammation, or steatosis could be observed [114], suggesting the need for longer treatment to achieve beneficial effects.

In patients with NAFLD, ameliorated levels of AST (aspartate transaminase), ALT (alanine transaminase), and γ-GT (gamma-glutamyl transferase) (50 mg/day for 4 months, 30 patients and 50 mg/day to 100 mg/day for 36 weeks, 36 patients) [115,116] and reduced intrahepatic lipid content and body fat (50 mg/day for 24 weeks, 20 overweight patients with T2DM) [117] were observed in three clinical trials. In another clinical trial, sitagliptin administrated as an add-on to metformin to patients with T2DM under inadequate glycemic control and NAFLD led to reduced intrahepatic lipid and visceral adipose tissue (100 mg/day, 26 weeks, 27 patients) [118]. On the other hand, in several clinical trials conducted on NAFLD patients, no significant decreases in liver transaminases [119,120,121,122,123] or hepatic fat content [123,124,125] were observed during treatment.

Regarding the other DPP-4 inhibitors, only a few clinical trials in humans have been conducted. Specifically, vildagliptin was found to be able to decrease ALT, AST, and fatty liver (50 mg twice daily for 12 weeks, 58 NAFLD patients) [126] as well as hepatic triglycerides (50 mg twice daily for 6 months, 44 T2DM patients) [127]. Alogliptin was only tested in one single arm, non-randomized, multi-center study indicating the ability to improve NASH in a small number of patients (25 mg/day, 39 NAFLD T2DM patients, 12 months) [128], and saxagliptin could improve hepatic steatosis (5 mg/day, 95 NAFLD T2DM patients, 24 weeks) [129].

Overall, these results suggest that the effect of DPP-4 inhibitors in resolving hepatic steatosis is limited, and randomized controlled clinical trials with increased numbers of participants and suitable durations are required in order to establish their role in NAFLD/NASH. Currently, there is only one ongoing clinical trial regarding DPP-4 inhibitors in NAFLD/NASH (NCT05195944) comparing the effectiveness of semaglutide with sitagliptin on glycemic control, body weight, safety, and tolerability in liver transplant recipients with poorly controlled diabetes mellitus.

### 7.2. Effect of GLP-1RAs and DPP-4i in CVDs

Most of the clinical trials that have been conducted to investigate the effect of GLP-1RAs in CVDs were performed in patients with T2DM. This is attributed to the fact that diabetes can lead to CVD but is also due to the fact that since 2008 it is a regulatory requirement for new antidiabetic medications to not only improve control over the blood glucose level but also to reduce the risk of both micro- and macrovascular complications. Hence, many CVOTs have been conducted or are still ongoing to prove the safety of new antidiabetic treatments in terms of CVD [130]. Ever since, the CVOTs that have been conducted on GLP-1 RAs indicate the non-inferiority of these drugs compared to placebo in terms of safety and, in some cases, their superiority in terms of risk reduction for the occurrence of CVD, i.e., beneficial effects on macro- or micro-vascular complications. These actions are not only attributed to the glycemic control exerted by these drugs but also to their ability to act on GLP-1 receptors on different body tissues (e.g., vascular, heart, muscle, peripheral nervous system, and immune cells). Moreover, cardiovascular safety is also found to be negatively related to the hypoglycemia that can be caused by some antidiabetic medications. As a result, good glycemic control that can diminish the possibility of hypoglycemic episodes can be extremely beneficial for avoiding CVD complications [13].

Most of the CVOTs conducted on GLP-1RAs use the first occurrence of a major adverse cardiovascular event (MACE), such as a non-fatal stroke, non-fatal myocardial infraction, hospitalization, or CVD-related death, as the primary endpoint. These studies were different in terms of the total duration, the number of enrolled patients, and the percentage of patients with pre-existing CVD (high- or low-risk populations) [13]. Based on the CVOTs conducted so far, liraglutide, semaglutide, dulaglutide, and albiglutide were found to significantly decrease the 3-point MACE, thus indicating their beneficial effects and superiority compared to placebo. Moreover, changes in body weight, systolic blood pressure (SBP), mean HbA_1(c)_ levels, and cholesterol levels in the groups receiving GLP-1 RAs compared to the placebo groups were observed in the conducted CVOTs.

Specifically, the effect of **liraglutide** in CVD was studied in the LEADER trial, where 9340 patients were enrolled, out of whom 81% had pre-existing CVD, with a median follow-up of 3.8 years. Overall, the number of patients reaching MACE was lower for those receiving liraglutide (13% vs. 14.9% in the placebo group; hazard ratio, 0.87; 95% CI, 0.78 to 0.97, *p* = 0.01 for superiority). Moreover, reduction in the death rates from any cause (8.2% in liraglutide groups vs. 9.6% in placebo group; hazard ratio, 0.85; 95% CI, 0.74 to 0.97; *p* = 0.02) or cardiovascular causes (4.7% in liraglutide groups vs. 6.0% in placebo group; hazard ratio, 0.78; 95% CI, 0.66 to 0.93; *p* = 0.007) were observed, while no statistically significant difference was detected in non-fatal myocardial infarction, non-fatal stroke, or hospitalization for heart failure. During the clinical trial, patients receiving liraglutide exhibited lower body weight (−2.3 kg; 95% CI, −2.5 to −2.0), decreased systolic blood pressure (SBP) (−1.2 mm Hg; 95% CI, −1.9 to −0.5), and lower levels of HbA_1(c)_ (−0.40 percentage points; 95% CI, −0.45 to −0.34) compared to the placebo group [131].

Two major phase 3 CVOTs have been conducted regarding **semaglutide**, the SUSTAIN-6 trial (2016), where semaglutide was administered subcutaneously in doses of 0.5 mg or 1.0 mg once weekly, and PIONEER-6 (2019), where semaglutide was administered orally at a dose of 14 mg once daily. In the SUSTAIN-6 trial, 3297 patients were enrolled, with 83.0% having established CVD and a median follow up time of 2.1 years. The incidence of a non-fatal stroke was significantly lower in those receiving semaglutide (1.6% vs. 2.7% in the placebo group; hazard ratio, 0.61; 95% CI, 0.38 to 0.99; *p* = 0.0). Moreover, a significant decrease was observed in the occurrence of the primary composite cardiovascular outcome (6.6% vs. 8.9% in the placebo group; hazard ratio, 0.74; 95% CI, 0.58 to 0.95; *p* = 0.02 for superiority), demonstrating a reduced risk of MACE. This was attributed to the decreased rate of non-fatal stroke since there were no significant differences in non-fatal myocardial infraction (2.9% vs. 3.9% in placebo group; hazard ratio, 0.74; 95% CI, 0.51 to 1.08; *p* = 0.12) or death rate from CVD [132]. In the PIONEER-6 trial, 3183 patients were enrolled, with 84.7% having established CVD and a median follow up time of 1.3 years. A non-significant decrease in the occurrence of the primary outcome was observed for patients receiving oral semaglutide compared to those receiving placebo (3.8% vs. 4.8% in the placebo group; hazard ratio, 0.79; 95% CI, 0.57 to 1.11; *p* = 0.17 for superiority), indicating the non-inferiority of the drug, while the hazard ratio was similar to that obtained in the SUSTAIN-6 trial. The low rate of CVD events in this trial was attributed to its short duration [133]. Semaglutide administration also decreased body weight (−2.9 kg for those receiving 0.5 mg, −4.3 kg for those receiving 1.0 mg, and −3.4 kg for those receiving oral semaglutide; 95% CI; *p* < 0.001), SBP (−1.3 mm Hg for those receiving 0.5 mg, −2.6 mm Hg for those receiving 1.0 mg, and −2.6 for those receiving oral semaglutide; 95% CI; *p* < 0.001), and HbA_1(c)_ levels (−0.7 for those receiving 0.5 mg, −1.0 for those receiving 1.0 mg, and −0.7 for those receiving oral semaglutide; 95% CI; *p* < 0.001). Additionally, subcutaneous administration of 1.0 mg semaglutide increased the levels of HDL cholesterol (1.01 ratio to baseline vs. 0.97 in the placebo group; 95% CI; *p* < 0.001) (no significant changes were observed regarding total cholesterol, LDL cholesterol, or triglycerides) [132,133].

**Dulaglutide’s** beneficial effect on CVD events in T2DM patients was observed in the phase 3 REWIND trial. In this trial, a total of 9901 patients were enrolled, with 31.5% having established CVD and a median follow up time of 5.4 years. Hence, this trial had the longest duration and lowest percentage of patients with pre-existing CVD (low-risk population) compared to the other CVOTs in GLP1-RAs. A significant reduction in the primary outcome (3-point MACE) was observed in patients receiving dulaglutide subcutaneously in a weekly injection of 1.5 mg (12% vs. 13.4% in the placebo group; hazard ratio 0.88; 95% CI; 0.79–0.99; *p* = 0.026), which was attributed to the significantly lower incidence of non-fatal stroke (2.7% vs. 3.5% in the placebo group; hazard ratio 0.76; 95% CI; 0.62–0.94; *p* = 0.017). No significant difference was observed in the death rate from any cause (10.8% vs. 12.0% in the placebo group; hazard ratio 0.90; 95% CI; 0.80–1.01; *p* = 0.067). Dulaglutide also reduced body weight (−1.46 kg; 95% CI, −1.67 to −1.25; *p* < 0.0001), SBP (−1.70 mm Hg; 95% CI, −2.07 to −1.33; *p* < 0.0001), HbA_1(c)_ levels (−0.61%; 95% CI, −0.65 to −0.58; *p* < 0.0001), total cholesterol levels (−0.07 mmol/L; 95% CI; −0.10 to −0.03; *p* = 0.0002), and LDL cholesterol (−0.05 mmol/L; 95% CI; −0.08 to −0.02; *p* = 0.001) [134].

The superiority of **albiglutide** compared to the placebo for the primary outcome of the first occurrence of a 3-point MACE was established in the HARMONY trial, which enrolled 9463 patients, with 100% having pre-existing CVD (high-risk population) and a median follow-up of 1.6 years. Albiglutide was administered subcutaneously (30–50 mg once a week) and exhibited a significant lowering of the incidence rate of MACE (7% vs. 9% in the placebo group; hazard group 0.78; 95% CI; 0.68–0.90; *p* = 0.0006 for superiority) [135]. Albiglutide’s ability to decrease body weight (−0.83 kg; 95% CI, −1.06 to −0.60) and SBP (−0.67 mm Hg; 95% CI, −1.40 to −0.06) was lower than those observed for liraglutide, semaglutide, and dulaglutide. Similarly, HbA_1(c)_ levels were decreased (−0.52%; 95% CI, −0.58 to −0.45) [136]. Albiglutide is no longer being marketed after a decision from GlaxoSmithKline to permanently discontinue the product for commercial reasons.

CVOTs conducted on **lixisenatide** (ELIXA) and **exenatide** (EXCEL) indicated that these drugs were not superior in terms of MACE occurrence compared to placebo but met the criteria of non-inferiority. Specifically, in the ELIXA trial 6068 patients were enrolled, all with pre-existing CVD, with a median follow-up time of 2.1 years. Lixisenatide was administered subcutaneously (10–20 μg daily) and exhibited a 13.4% occurrence of the primary endpoint compared to the 13.2 % obtained in the placebo group (hazard ratio, 1.02; 95% CI; 0.89 to 1.17; *p* < 0.001 for non-inferiority and *p* = 0.81 for superiority). No significant differences were observed in terms of hospitalization rates for heart failure or death rates. HbA_1(c)_ levels were reduced (−0.27%; 95% CI, −0.31 to −0.22; *p* < 0.001). Body weight and SBP were moderately decreased (−0.7 kg; 95% CI, −0.9 to −0.5; *p* < 0.001 and −0.8 mmHg; 95% CI, −1.3 to −0.3; *p* = 0.001, respectively) [137]. In the EXCEL trial, 14,752 patients enrolled, making this the largest study of all the CVOTs performed with GLP-1 RAs. Out of these patients, 73.1% had a pre-existing CVD, and the trial had a median follow-up of 3.2 years. Exenatide was administered as 2 mg once weekly. The results indicated 11.4% with MACE occurrence in the group receiving exenatide, while 12.2% was observed in the placebo group (hazard ratio, 0.91; 95% CI, 0.83 to 1.00; *p* < 0.001 for noninferiority and *p* = 0.06 for superiority). There were no significant changes in myocardial infraction or stroke (fatal or non-fatal), hospitalization for heart failure or acute coronary syndrome, or death from cardiovascular causes. Similar reductions in HbA_1(c)_ levels (−0.53%; 95% CI, −0.57 to −0.50; *p* < 0.001), body weight (−1.27 kg; 95% CI, −1.40 to −1.13; *p* < 0.001), and SBP (−1.57 mm Hg; 95% CI, −1.92 to −1.21; *p* < 0.001) were observed. Additionally, LDL was lower in the exenatide group compared to the placebo group (−0.04 mmol/L; 95% CI, −0.06 to −0.01; *p* = 0.004) [138].

A meta-analysis of CVOTs conducted on GLP-1 RAs, which did not include results from the REWIND trial, indicated that they can decrease the risk of MACE by 14% (hazard ratio 0.86; 95% CI, 0.80–0.93; *p* = 0.002) in patients with pre-established atherosclerotic CVD (no effect was observed in diabetic patients without such a background). Moreover, GLP-1 RAs did not significantly affect hospitalization for heart failure but were able to decrease the risk of non-fatal stroke as well as the risk of kidney disease development, including macroalbuminuria [139].

In terms of adverse effects, higher percentages of occurrence leading to drug discontinuation compared to placebo were reported for liraglutide, semaglutide (higher percentage was observed in subcutaneous administration of 1 mg semaglutide), and lixisenatide. These adverse events were mainly gastrointestinal disorders, with those occurring more often being nausea, vomiting, and diarrhea. The occurrence of serious adverse events, overall cancer, acute pancreatitis, benign or malignant neoplasms, pancreatic cancer, medullary thyroid carcinoma, and severe hypoglycemia did not differ significantly between groups (drug vs. placebo) for all the tested drugs.

Based on the results obtained from the various CVOTs, the administration of GLP-1 RAs with proven beneficial effects (i.e., liraglutide, semaglutide, and dulaglutide) is recommended by the American Diabetes Association (ADA) and the European Association for the study of Diabetes (EASD) for patients with T2DM and pre-established atherosclerotic CVD or at a high CV risk, where the benefit of these drugs is more enhanced [140].

Regarding the effect of GLP-1 RAs on MACE in T2DM patients of different races (Whites, Blacks, Asians, and others), in a metanalysis performed by Mei Qiu et al. (2020) on CVOTs of GLP-1 RAs it was found that GLP-1 RAs reduced the risk of MACE in all race subgroups with the exception of the Black race (0.92; 95% CI, 0.70–1.20; *p* < 0.05) [141]. These results were in accordance with another systematic review and meta-analysis that was performed by Mishriky et al. (2019) regarding the Black race on CVOTs of GLP-1 RAs and DPP-4i, in which only a small percentage of diabetic patients enrolled in the CVOTs were Black (4.5%), and there was no significant difference between the placebo and drug groups in Black patients in terms of MACE incidence (0.94; 95% CI, 0.77, 1.16; *p* < 0.05) [142]. Nevertheless, since data are limited, to draw safer conclusions additional more targeted clinical trials in specific race groups need to be conducted. Furthermore, NAFLD prevalence, as well as the risk of NASH amongst NAFLD patients, is lower in Blacks than Whites [143].

For DPP-4 inhibitors, no clinical trial so far has shown beneficial effects in CVD. Saxagliptin was not found to be superior versus the placebo in patients with pre-established CVD or at a high risk (SAVOR-TIMI53), whereas alogliptin was found to be non-inferior (EXAMINE) [144].

Various clinical trials are ongoing for the assessment of the effectiveness of GLP-1 RAs, especially semaglutide, liraglutide and exenatide, on CVD and atherosclerosis in patients with or without T2DM and obesity (Table 2).

## 8. Explaining the Effectiveness of Incretin-Based Drugs on NASH and Atherosclerosis

### 8.1. Potential Mechanisms of GLP-1 RAs’ Effectiveness in NASH

**Figure 2:**
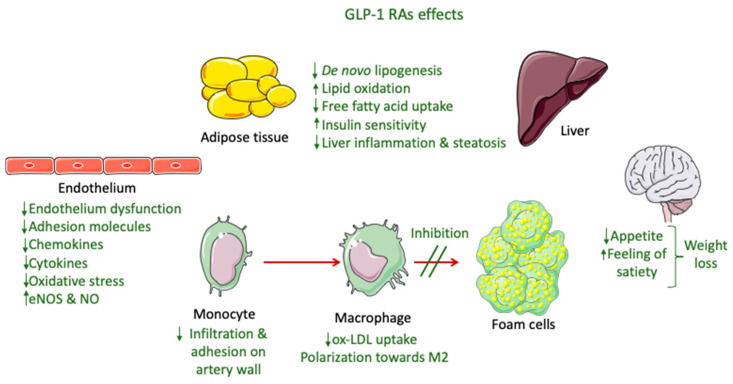
GLP-1 RA effects involved in NASH and atherosclerosis improvement.

The only GLP-1 RAs that have been proven to resolve NASH based on histological data are liraglutide and semaglutide. These GLP-1 RAs, apart from their glucose-lowering effect, have demonstrated an ability to significantly reduce body weight, with semaglutide having a more pronounced effect. This effect is mainly attributed to a modulation of appetite and a feeling of satiety as well as reduced caloric intake through actions in the central nervous system combined with a reduction in glucosuria due to enhanced glycemic control (Figure 2) [145]. Most of the weight that is being lost during treatment with GLP-1 RAs is fat mass, particularly visceral fat, due to their effect on adipose tissue [146]. However, although body weight reduction is a key parameter in NASH resolution, it cannot solely explain the improved liver function observed in patients treated with GLP-1 RAs. Specifically, in a study performed by Shiomi et al. (2020) in Japanese patients with T2DM and NAFLD receiving liraglutide for 24 weeks the improvement in liver function or fibrosis (assessed through aspartate aminotransferase, alanine aminotransferase, and fibrosis-4 indices) was found to be independent of the body mass index [147]. Several other mechanisms are involved, as demonstrated in Figure 2.

Preclinical studies indicate that the reduced hepatic steatosis is attributed to more direct actions of GLP-1 RAs to hepatocytes, through the modulation of lipid metabolism, i.e., reduced fatty acid uptake and de novo lipogenesis, and enhanced lipid oxidation. Nevertheless, the presence of GLP-1 receptors in hepatocytes is still being questioned [148]. The increase in insulin sensitivity in hepatic and adipose tissue leads to lipolysis suppression, which is attributed to the augmented action of insulin. This, in turn, decreases the de novo lipogenesis in hepatocytes [149]. Moreover, an increase in the adiponectin/leptin ratio caused by an enhancement of insulin action in peripheral tissues produces reduced liver inflammation [148]. Liraglutide has also exhibited in vitro anti-inflammatory liver effects, through promoting mitophagy for the elimination of dysfunctional mitochondria, thus suppressing the NLRP3 inflammasome as well as the pyroptotic death of hepatocytes [150]. GLP-1 RAs also protect hepatocytes from apoptosis related to fatty acids by blocking a dysfunctional endoplasmic reticulum stress response [151]. Furthermore, exenatide demonstrated an ability to decrease the lipid content and inflammation in the liver of APOE*3-Leiden.CETP mice by inhibiting the expression of liver chemokines and the gathering of oxLDL in macrophages [152]. GLP-1 RAs activity on both hepatic and adipose tissue, which is linked with suppressed expression of pro-inflammatory mediators, such as cytokines (e.g., IL-6 and TNF-a) and chemokines (e.g., monocyte chemoattractant protein-1 (MCP-1), vascular cell adhesion molecule (VCAM-1), intercellular adhesion molecule-1 (ICAM-1), and E-selectin), is fundamental in regulating fibrosis in NASH [153,154]. Moreover, this downregulation, along with an inhibition of macrophage infiltration, plays an important role in enhancing insulin sensitivity. These effects have been linked with the suppression of nuclear factor κB (NF-κB) and the upregulation of IκB kinase produced by GLP-1 RAs as well as interference with the c-Jun NH2-terminal kinase (JNK) pathway, which is related to averting beta cell apoptosis [154].

### 8.2. Potential Mechanisms of GLP-1 RAs’ Effectiveness in Atherosclerosis

The beneficial cardiovascular effect of GLP-1 RAs that has been demonstrated in CVOTs is mainly associated to the prevention of atherosclerotic events, especially in a decrease in non-fatal strokes and non-fatal myocardial infractions. These effects cannot be attributed only to the activity of GLP-1 RAs on controlling glucose levels and reducing body weight [134]. Multiple actions on inflammation, lipid levels, vascular smooth muscle cells (VSMC), and endothelium seem to be involved (Figure 2), with the exact mechanisms still under investigation [139].

A meta-analysis performed to examine the effects of GLP-1 RAs in atherosclerosis demonstrated their beneficial role through a decrease in the blood levels of plasminogen activator inhibitor-1 (PAI-1), high-sensitivity c-reactive protein (hsCRP), and brain natriuretic peptide (BNP), all of which are atherosclerosis markers, as well as total and LDL cholesterol and triglycerides [155].

A study on the cardio-metabolic effect of liraglutide in T2DM patients with MetS, with a total follow-up time of 18 weeks and no control group, showed that the drug (in combination with metformin) was able to decrease the carotid intima–media thickness (cIMT), a biomarker of subclinical atherosclerosis, as well as MetS prevalence, with a significant association between these two being identified. These results were seen early in the study (only 6 months after treatment initiation) [156]. Additionally, total and LDL cholesterol as well as triglycerides were found to be reduced during treatment [156], although a later post hoc analysis for the LEADER trial suggested that liraglutide’s benefits are not related to LDL-C levels since they were evident, even in the very low baseline LDL-C [157]. Recently, liraglutide was found to decrease the atherogenic small dense LDL-3 and LDL-4 subfractions of LDL cholesterol, with the former being correlated with a reduction in cIMT, which further enhances the theory that GLP-1 RAs’ effect is exerted separately from their glycemic and weight control effect [158].

A recent clinical study performed by Yang et al. (2021) separated the beneficial CV effect of GLP-1 RAs in terms of atherosclerosis progression from their hypoglycemic effect in patients with coronary heart disease (CHD), indicating that their role is associated with the polarization of macrophages towards M2, i.e., the anti-inflammatory type (Figure 2) [159]. The anti-inflammatory effect of liraglutide has also been previously reported from preclinical studies [160]. Specifically, liraglutide’s effect in pre-established atherosclerosis has been studied in vivo in ApoE^−/−^ mice and ex vivo in human atherosclerotic plaques, with the results indicating the drug’s ability to reduce M1 proinflammatory mediators, such as MCP-1, TNF-a, and IL-1b, and upregulate the cathepsin protein family in the bone marrow, leading to an attenuation of atherosclerosis in the aorta through an increase in M2-like macrophages [160]. Changes in macrophages towards the M2 phenotype were also reported for exenatide based on in vitro experiments [161].

Liraglutide has also been found to improve plaque stability and hinder atherosclerotic plaque development in apolipoprotein-E-deficient (ApoE^−/−^) mice by suppressing endothelial dysfunction and the expression of vascular adhesion molecules (Figure 2) [162]. Similar results were obtained in another study performed by Rakipovsi et al. (2018) in ApoE^−/−^ and low-density lipoprotein receptor-deficient (LDLr^−/−^) mice, demonstrating that liraglutide and semaglutide significantly lessened the development of plaque lesions, mainly due to anti-inflammatory effects. In this case, reductions in body weight and cholesterol levels were only partially involved, taking into consideration that the model used in that study was non-diabetic. These results were based on the observed reduction in the blood levels of systemic inflammation markers (TNF-a and interferon-γ) and the downregulation of various inflammatory pathways based on a transcription analysis of aortic atherosclerotic tissue (modified gene expression of proteins involved in leucocyte recruitment, adhesion, and migration) [163]. Additionally, liraglutide administered in ApoE^−/−^ mice was found to exert its protective role mainly through down-regulating ACAT1 (responsible for producing cholesterol ester from free cholesterol), accompanied by the upregulation of ATP-binding cassette transporter A1 (ABCA1) (responsible for the efflux of free cholesterol) and the downregulation of scavenger receptor CD36 (responsible for uptake of oxLDL), which are involved in the monocyte/macrophage infiltration in the walls of arteries and the transformation of macrophages into foam cells [164].

Plaque stability has also been linked with the ability of GLP-1 RAs to modify the levels of matrix metalloproteinases (MMPs) and tissue inhibitors of metalloproteinases (TIMPs). MMPs can degrade the extracellular matrix, leading to intima thickening and vascular remodeling, and are modulated by TIMPs. Exenatide was found to regulate the in vitro expression of both MMPs and TIMPs in human coronary artery and aortic endothelial cells as well as coronary artery smooth muscle cells by suppressing NF-κB and Akt-Thr308 phosphorylation [165,166]. Similar results were obtained for liraglutide in C57BL/6J mice, with a reduced expression of MMP-9 as well as MCP-1 and ICAM-1, which were attributed to the exerted regulation of the Akt and extracellular signal-regulated kinase (ERK) pathways [167].

Additionally, exenatide was found to decrease oxidative stress (by suppressing the expression of NADPH oxidase, which is involved in ROS production) and ameliorate antioxidative potential (by enhancing the expression and activity of antioxidative enzymes SOD and GSH-Px) in human macrophages, processes that are involved in the pathogenesis and acceleration of atherosclerosis [168].

The protective role of GLP-1 RAs, specifically dulaglutide, in the endothelium regarding atherosclerosis was demonstrated in preclinical studies in human aortic endothelial cells (HAECs). Dulaglutide was found to prevent the atherosclerotic effects induced by oxLDL by preventing p53 protein phosphorylation and thus averting the downregulation of Krüppel-like Factor 2 (KLF2), a factor that greatly contributes to vascular endothelial cell protection by inhibiting monocyte adhesion to the endothelium and promoting nitric oxide synthase expression by endothelial cells (eNOS). Hence, dulaglutide hindered the production of proinflammatory cytokines and chemokines (such as IL-1β, IL-6, MCP-1, and high-mobility group protein 1 (HMGB-1)) as well as molecules that induce the adhesion of monocytes to endothelial cells, especially VCAM-1 and E-selectin [169]. Similar observations were made for luraglutide on KLF2, where its protective effect was found to be dependent on ERK-5 [170]. Exenatide was also found to decrease the recruitment of macrophages from the circulation and adhesion to the vessel wall [152].

The direct protective anti-inflammatory effect of GLP-1 RAs on the endothelium has also been elucidated from in vivo experiments in non-diabetic hypertensive mice, where liraglutide was able to decrease vascular inflammation and oxidative stress by averting endothelial NO synthetase (eNOS) uncoupling (thus inhibiting the production of ROS) and enhancing NO bioavailability, actions that are related to GLP-1 receptors in the endothelial cells but not on myeloid cells (Figure 2) [171]. Furthermore, liraglutide was found to obstruct platelet activation though in vitro experiments, a process that is involved in the pathogenesis of atherosclerosis by activating pathways that lead to NO production [172]. The protective action of GLP1-RAs on the endothelium, as well as their ability to reduce oxidative stress and, hence, endothelium disfunction and autophagy, has also been reported by other groups [173,174,175].

## 9. Conclusions

NAFLD is the hepatic manifestation of MetS and a risk factor for hypertension, dyslipidemia, and type 2 diabetes mellitus. The pathogenesis of NAFLD and the progression to NASH is a multifactorial process where hepatic lipotoxicity, inflammation, and oxidative stress play key roles. The resulting cellular injury triggers immune-mediated hepatocellular apoptosis/necrosis, leading to HSC activation and fibrogenesis [176]. Currently, there are no drugs approved for NAFLD/NASH treatment, and official directions focus on life-style changes, including exercise and weight loss. Since T2DM and the presence of MetS are associated with NAFLD/NASH development and progression, various antidiabetic medications, such as incretin-based drugs, have been examined for their effects in these conditions. Out of the incretin-based drugs tested for their ability to resolve NASH, only semaglutide and liraglutide, belonging to the class of GLP-1 RAs, were found to be effective so far, but no improvement in fibrosis was observed during the clinical trials, probably due to the duration of these trials being shorter than the time needed for more pronounced changes in the liver’s fibrotic state. Moreover, clinical trials that are conducted with the use of liver biopsy for NASH evaluation are rare, though they are more informative on the diagnosis and progression of the disease due to the more complicated implementation. As a result, clinical trials that are planned to have longer durations and/or evaluations of the effect in the liver through biopsy are expected to shed more light in the future regarding the possible effect of GLP-1 RAs in NASH and liver fibrosis.

The effectiveness of GLP-1 RAs in treating NASH is multifactorial, with many different mechanisms being involved. The induced weight loss as well as the glycemic control achieved by GLP-1 RAs are parameters with significant roles in NASH development and progression but cannot account on their own for the beneficial effects observed in the performed clinical trials. More direct effects on the hepatic and adipose tissue as well as anti-inflammatory properties are contributing factors. Specifically, there are indications that GLP-1 RAs can downregulate de novo lipogenesis, up-regulate lipid oxidation and free fatty acid uptake through an improvement in insulin sensitivity, and increase the levels of adiponectin by ameliorating insulin activity in peripheral tissue, leading to reduced liver inflammation [148,149]. Additional anti-inflammatory actions involve the suppression of the expression of liver chemokines and oxLDL uptake from macrophages as well as the induction of mitophagy of dysfunctional mitochondria and the blocking of the dysfunctional endoplasmic reticulum stress response, thus preventing hepatocyte apoptosis (Figure 1) [154,156,157].

On the other hand, atherosclerosis is a condition that develops quite often in patients with T2DM, with dysfunction in the endothelium and increased inflammation having a fundamental role. Many mechanisms are considered to be shared in T2DM and atherosclerosis development, such as oxidative stress, inflammation, and the modification of the extracellular matrix [177]. At the same time, patients with NASH and/or advanced fibrosis as well as NAFLD patients with concomitant T2DM have the highest risk for the development of atherosclerotic cardiovascular diseases [178]. Visceral and ectopic fat accumulation in NASH is associated with an over-secretion of FFAs and a number of adipocytokines, including TNF-a, IL-6, and PAI-1, promoting vascular inflammation and endothelial dysfunction, a marker of early atherosclerosis. Insulin resistance, atherogenic dyslipidemia, and lipotoxicity promote oxidative stress with the concomitant activation of inflammatory pathways and the release of procoagulant factors, further contributing to the acceleration of the atherosclerotic process (Figure 1) [179].

Many CVOTs that have been conducted on GLP-1 RAs have shown that liraglutide, semaglutide, and dulaglutide can exert a beneficial role in CVD, mainly due to the effect of these drugs in preventing atherosclerotic events. These effects are attributed partly to the glycemic control, weight loss, and subsequent reduction in total cholesterol and triglycerides produced by GLP-1 RAs and also to their anti-inflammatory properties. The latter are related to an inhibition of the expression of pro-inflammatory mediators, such as chemokines and cytokines, as well as a suppression of oxidative stress and endothelium dysfunction, inhibition of monocyte/macrophage infiltration and adhesion, macrophage polarization towards M2, and an inhibition of macrophage transformation to foam cells, all leading to improved plaque stability and impeding plaque progression (Figure 1) [159,160,162,163,164,168,169,170].

Overall, inflammation is a key component for the development of both NASH and atherosclerosis. Hence, the anti-inflammatory properties of GLP-1 RAs, as summarized in this review, are fundamental for the simultaneous treatment of both diseases. The drugs’ additional ability to reduce weight and affect lipid oxidation, uptake, and lipogenesis synthesize a unique and valuable activity profile. While presently many clinical data exist on the separate beneficial effects of GLP-1 RAs on NASH resolution and atherosclerosis development and progression, there is a lack of clinical trials for the evaluation of the simultaneous effectiveness of these drugs in patients suffering from both NASH and atherosclerosis. Nevertheless, the data that have been collected and analyzed in this review provide a strong indication of the ability of GLP-1 RAs, especially liraglutide and semaglutide, to simultaneously treat NASH and inhibit the progression of atherosclerosis, thus protecting against atherogenic cardiovascular events.

## Figures and Tables

**Table 1:** Ongoing clinical trials for GLP-1 RAs on nonalcoholic steatohepatitis (NASH).

Clinical Trial Title/NCT Number	Condition	Intervention	Primary Outcome	Phase	Duration
Researching an Effect of GLP-1 Agonist on Liver STeatosis (REALIST)/NCT03648554	T2DM, NASH	dulaglutide (TRULICITY^®^) 1.5 mg	Histological improvement (based on NAS score) without worsening of fibrosis (based on liver biopsy)	4	Treatment: 52 weeks Follow-up: 24 weeks
Research Study on Whether Semaglutide Works in People with Non-alcoholic Steatohepatitis (NASH)/NCT04822181	NASH	Semaglutide	Resolution of steatohepatitis and no worsening of liver fibrosis (based on NAS)/Improvement in liver fibrosis and no worsening of steatohepatitis (based on NAS)/Time to first liver-related clinical event	3	72 weeks/72 weeks/240 weeks
Study Evaluating the Safety and Efficacy of Semaglutide, and the Fixed-Dose Combination of Cilofexor and Firsocostat, Alone and in Combination, in Participants With Compensated Cirrhosis (F4) Due to Nonalcoholic Steatohepatitis (NASH)/NCT04971785	NASH	Semaglutide (SEMA)/Cilofexor (CILO)/Firsocostat (FIR)	Improvement in fibrosis/NASH resolution (based on NAS)	2	72 weeks
Research Study on Whether a Combination of 2 Medicines (NNC0194 0499 and Semaglutide) Works in People with Non-alcoholic Steatohepatitis (NASH)/NCT05016882	NASH	NNC0194-0499 and semaglutide	Improvement in liver fibrosis and no worsening of NASH (based on NAS)	2	52 weeks
Non-Alcoholic Fatty Liver Disease, the HEpatic Response to Oral Glucose, and the Effect of Semaglutide (NAFLD HEROES)/NCT03884075	NASH/NAFLD	Semaglutide	Histological improvement (based on NAS)/Clinical improvement/Change in hepatic gene expression	2	30 weeks
Combined Active Treatment in Type 2 Diabetes with NASH (COMBAT_T2_NASH)/NCT04639414	T2DM, NASH, NAFLD	Empagliflozin/Semaglutide	Histological resolution of NASH without worsening of fibrosis (based on NAS)	4	48 weeks

**Table 2:** Ongoing clinical trials for GLP-1 RAs on Atherosclerosis and Cardiovascular Diseases.

Clinical Trial Title/NCT Number	Condition	Intervention	Primary Outcome	Phase	Duration
Efficacy and Safety of Liraglutide in Type 2 Diabetes with Lower Extremity Arterial Disease/NCT04146155	T2DM, Peripheral Vascular Disorder Due to Diabetes Mellitus	Liraglutide + standard-of-care treatment	Initial and absolute claudication distance	4	24 weeks
Liraglutide and Peripheral Artery Disease (STARDUST)/NCT04881110	T2DM, Peripheral Arterial Disease	Liraglutide	Peripheral transcutaneous oxygen pressure	4	6 months
Effects on Re-endothelialisation with Bydureon Treatment in Type 2 Diabetes Subjects/NCT02162550	Atherosclerosis Diabetes Restenosis	Exenatide	The degree of non-covered stent struts analyzed by optical coherence tomography	4	12 weeks
Effect of Semaglutide in Coronary Atheroma Plaque/NCT05071417	Atherosclerosis, Coronary Artery Disease	Semaglutide	Plaque burden modification assessed by coronary CT and plaque quantification	3	18 months
Semaglutide Treatment on Coronary Progression/NCT03985384	T2DM, Coronary Artery Disease	Semaglutide	Rate of change in non-calcified plaque volume	4	12 months
A Research Study of How Semaglutide Works in People with Disease Affecting the Heart and/or Blood Vessels and Type 2 Diabetes/NCT04032197	T2DM	Semaglutide	Change in maximum target-to-background ratio for 18F-fluorodeoxyglucose in the carotid arteries	1	26 weeks
A Research Study to Compare a Medicine Called Semaglutide Against Placebo in People with Peripheral Arterial Disease and Type 2 Diabetes/NCT04560998	T2DM, Peripheral Arterial Disease	Semaglutide	Change in maximum walking distance on a constant load treadmill test	3	52 weeks
Study of Semaglutide for Non-Alcoholic Fatty Liver Disease (NAFLD), a Metabolic Syndrome with Insulin Resistance, Increased Hepatic Lipids, and Increased Cardiovascular Disease Risk (The SLIM LIVER Study)/NCT04216589	HIV Infections, NAFLD	Semaglutide	Change (absolute) in intra-hepatic triglyceride content (%)	2	24 weeks
Research Study to Look at How Well Semaglutide Works in People Living with Heart Failure, Obesity and Type 2 Diabetes/NCT04916470	Heart Failure with Preserved Ejection Fraction (HFpEF) and T2DM	Semaglutide	Change in Kansas City Cardiomyopathy Questionnaire clinical summary score, change in body weight	2	52 weeks
Semaglutide for the Reduction of Arrhythmia Burden in Overweight AF Patients/NCT04885634	Atrial Fibrillation, Overweight and Obesity	Semaglutide	Number of participants to complete recruitment and complete follow-up, total resource requirement	3	75 weeks
Research Study to Investigate How Well Semaglutide Works in People Living with Heart Failure and Obesity/NCT04788511	T2DM, Peripheral Arterial Disease	Semaglutide	Change in maximum walking distance on a constant load treadmill test	3	52 weeks
Comparison of Type 2 Diabetes Pharmacotherapy Regimens/NCT05073692	T2DM, Cardiovascular Diseases	Linagliptin Exenatide Liraglutide Empagliflozin Glimepiride Glipizide	Incidence of 3-point major adverse cardiovascular events (MACE)	Observational	
Liraglutide Effect in Atrial Fibrillation/NCT03856632	Atrial Fibrillation	Liraglutide	Change in size of left atrial epicardial adipose tissue	4	3 months (prior to ablation)
Incretin and Treatment with Inhibition of Sodium-glucose Cotransporter-2 Combination Insights into Mechanisms Implicated in Congestive Heart Failure: “NATRIURETIC” Trial/NCT04535960	T2DM	Liraglutide + Empagliflozin	Proximal tubular natriuresis	2	12 weeks

## Abbreviations

AASLD	American Association for the Study of Liver Diseases
ACAT1	acyl-CoA: cholesterol acyltransferase 1
ADA	American Diabetes Association
AFLD	alcoholic fatty liver disease
ALT	alanine aminotransferase
ApoE^−/−^	apolipoprotein E-deficient
ASH	alcohol-related steatohepatitis
BNP	brain natriuretic peptide
CAD	coronary artery disease
CI	confidence interval
CIDEB	cell death-inducing DFFA-like effector b
cIMT	carotid–intima media thickness
CK-18	cytokeratine-18
CRP	C-reactive protein
CT	computed tomography
CV	cardiovascular
CVD	cardiovascular diseases
CVOTs	cardiovascular outcome trials
DAMPs	damage-associated molecular patterns
DNL	de novo lipogenesis
DPP-4i	dipeptidyl dipeptidase-4 inhibitors
EASD	European Association for the study of Diabetes
EASL	European Association for the Study of Liver
ECs	endothelial cells
eNOS	endothelium nitric oxide synthase
ER	endoplasmic reticulum
ERK	extracellular signal-regulated kinase
FAO	fatty acid β-oxidation
FDA	Food and Drug Association
FFAs	free fatty acids
FibroScan	transient elastography
FOXO1	forkhead transcription factor O1
FXRs	farnesoid X receptors
GCKR	glucokinase regulator
GLP-1 RAs	glucagon-like peptide-1 receptor agonists
GPAM	glycerol-3-phosphate acyltransferase
GWAS	genome-wide association studies
HAECs	human aortic endothelial cells
HbA_1(c)_	glycated hemoglobin
HMGB-1	high-mobility group protein 1
hsCRP	high-sensitivity c-reactive protein
HSCs	hepatic stellate cells
17β-HSD13	hydroxysteroid 17-beta dehydrogenase 13
ICAM-1	intercellular adhesion molecule-1
IL	interleukin
JNK	c-Jun NH2-terminal kinase
KLF2	Krüppel-like Factor 2
LDLr^−/−^	low-density lipoprotein receptor deficient
LOX-1	lectin-like oxLDL receptor-1
Lp(a)	lipoprotein (a)
MACE	major adverse cardiovascular event
MBOAT7	membrane-bound O-acyltransferase domain-containing 7
MCP-1	monocyte chemoattractant protein-1
MetS	metabolic syndrome
MMPs	matrix metalloproteinases
MRI	magnetic resonance imaging
NAFLD	nonalcoholic fatty liver disease
NAS	NAFLD activity score
NASH	nonalcoholic steatohepatitis
NASH CRN	NASH Clinical Research Network
NCEH	neutral cholesterol ester hydrolase
NK	natural killer
NLRP3	NOD-like receptor protein 3
NLRs	NOD-like receptors
oxLDL	oxidized low-density lipoprotein
PAI-1	plasminogen activator inhibitor-1
PAMPs	pathogen-associated molecular patterns
PDFF	proton density fat fraction
PDGF	platelet-derived growth factor
PNPLA3	patatin-like phospholipase domain-containing 3
PRRs	pattern recognition receptors
PUFAs	polyunsaturated fatty acids
PYGO1	Pygopus family PHD finger 1
ROS	reactive oxygen species
SCFAs	short-chain fatty acids
SR	scavenger receptor
T2DM	type 2 diabetes mellitus
TGF-β1	transforming growth factor
TGR5	G-protein-coupled receptor 5
TGs	triglycerides
TIMP-1	tissue inhibitor of metalloproteinases 1
TIMPs	tissue inhibitor of metalloproteinases
TLRs	toll-like receptors
TM6SF2	transmembrane 6 super-family member 2
TNF-a	tumor necrosis factor-a
VCAM-1	vascular cell adhesion molecule
VLDL	very low density lipoproteins
VSMC	vascular smooth muscle cells
